# Negative symptoms in First-Episode Schizophrenia related to morphometric alterations in orbitofrontal and superior temporal cortex: the OPTiMiSE study

**DOI:** 10.1017/S0033291722000010

**Published:** 2023-06

**Authors:** Arsime Demjaha, Silvana Galderisi, Birthe Glenthøj, Celso Arango, Armida Mucci, Andrew Lawrence, Owen O'Daly, Matthew Kempton, Simone Ciufolini, Lone Baandrup, Bjørn H. Ebdrup, Roberto Rodriguez-Jimenez, Maria Diaz-Marsa, Covadonga Martinez Díaz-Caneja, Inge Winter van Rossum, Rene Kahn, Paola Dazzan, Philip McGuire

**Affiliations:** 1 Department of Psychosis Studies, Institute of Psychiatry, Psychology & Neuroscience, King's College London, London, UK; 2 National Institute for Health Research Mental Health Biomedical Research Centre at South London and Maudsley NHS Foundation Trust and King's College London, London, UK; 3 Department of Psychiatry, University of Campania Luigi Vanvitelli, Caserta, Italy; 4 Faculty of Health and Medical Sciences, Department of Clinical Medicine, Center for Clinical Intervention and Neuropsychiatric Schizophrenia Research, CINS, Mental Health Center Glostrup, University of Copenhagen, Copenhagen, Denmark; 5 Department of Child and Adolescent Psychiatry, Institute of Psychiatry and Mental Health. Hospital General Universitario Gregorio Marañón. IiSGM, CIBERSAM. School of Medicine, Universidad Complutense de Madrid, Madrid, Spain; 6 Department of Neuroimaging, Institute of Psychiatry, Psychology, and Neuroscience, King's College London, London, UK; 7 Department of Psychiatry, Brain Center Rudolf Magnus, Utrecht, Netherlands; 8 Department of Psychiatry, Icahn School of Medicine at Mount Sinai, New York, NY, USA

**Keywords:** Cortical thickness, first-episode psychosis, FreeSurfer, negative symptoms, voxel-based morphometry

## Abstract

**Background:**

Negative symptoms are one of the most incapacitating features of Schizophrenia but their pathophysiology remains unclear. They have been linked to alterations in grey matter in several brain regions, but findings have been inconsistent. This may reflect the investigation of relatively small patient samples, and the confounding effects of chronic illness and exposure to antipsychotic medication. We sought to address these issues by investigating concurrently grey matter volumes (GMV) and cortical thickness (CTh) in a large sample of antipsychotic-naïve or minimally treated patients with First-Episode Schizophrenia (FES).

**Methods:**

T1-weighted structural MRI brain scans were acquired from 180 antipsychotic-naïve or minimally treated patients recruited as part of the OPTiMiSE study. The sample was stratified into subgroups with (*N* = 88) or without (*N* = 92) Prominent Negative Symptoms (PMN), based on PANSS ratings at presentation. Regional GMV and CTh in the two groups were compared using Voxel-Based Morphometry (VBM) and FreeSurfer (FS). Between-group differences were corrected for multiple comparisons via Family-Wise Error (FWE) and Monte Carlo z-field simulation respectively at *p* < 0.05 (2-tailed).

**Results:**

The presence of PMN symptoms was associated with larger left inferior orbitofrontal volume (*p* = 0.03) and greater CTh in the left lateral orbitofrontal gyrus (*p* = 0.007), but reduced CTh in the left superior temporal gyrus (*p* = 0.009).

**Conclusions:**

The findings highlight the role of orbitofrontal and temporal cortices in the pathogenesis of negative symptoms of Schizophrenia. As they were evident in generally untreated FEP patients, the results are unlikely to be related to effects of previous treatment or illness chronicity.

## Introduction

Negative symptoms are among the most incapacitating features of Schizophrenia, and are associated with particularly poor functional and clinical outcomes (Galderisi, Mucci, Buchanan, & Arango, 2018; Kirkpatrick, Fenton, Carpenter, & Marder, 2006). Whereas treatment with antipsychotic medication can reduce positive psychotic symptoms, it has relatively little effect on primary negative symptoms (Aleman et al., 2017; Fusar-Poli et al., 2015). This suggests that negative symptoms have a different pathophysiological basis to positive symptoms (Demjaha et al., 2014; Demjaha, Murray, McGuire, Kapur, & Howes, 2012). However, their neurobiological basis is still unclear.

The general inconsistency in findings to date may reflect between-study differences in methodology and design. Only a few studies have included large numbers of participants, and these may have been confounded by the effects of illness chronicity (Arango et al., 2012) and previous treatment. Furthermore, most neuroimaging studies of negative symptoms have examined grey matter volume (GMV) using voxel-based morphometry (VBM) (Benoit, Bodnar, Malla, Joober, & Lepage, 2012; Lacerda et al., 2007; Ren et al., 2013). VBM technique, while considerably advantageous is not able to differentiate between finer cortical structures. Cortical architecture is complex; its volume is composed of surface area and cortical thickness (CTh) that are phenotypically and genetically separable (Winkler et al., 2010). Although both these cortical indices impact on volume measurements of cortical grey matter, GMV appears to be more closely related to the surface area than CTh (Winkler et al., 2010). It has thus been suggested that investigating, in particular, CTh in addition to GMV, may provide additional and more sensitive information about underlying neuropathology of psychiatric disorders (Schultz et al., 2010). In addition measures of CTh are more sensitive than VBM and are thus complementary when defining GM anomalies (Bodnar et al., 2014). Thus, the concurrent use of both imaging techniques is increasingly advocated when investigating the pathophysiology of specific disease or cluster of Schizophrenia symptoms (Kong et al., 2015; Palaniyappan & Liddle, 2012). Recently with the advancement of neuroimaging techniques, the studies have employed new and more precise imaging software such as for instance FreSurfer, able to measure CTh (Walton et al., 2018; Xiao et al., 2015). However, all studies but one, that was limited by modest sample size (Venkatasubramanian, Jayakumar, Gangadhar, & Keshavan, 2008), have examined either GMV or CTh.

The minority of studies that have investigated relatively homogeneous samples and antipsychotic-naïve patients may have been limited by relatively small sample sizes and suboptimal assessments of negative symptoms. The way that negative symptoms were assessed and the brain regions of interest have been defined have varied largely between studies, (Lacerda et al., 2007) and some studies have only included male participants (Chemerinski, Nopoulos, Crespo-Facorro, Andreasen, & Magnotta, 2002; Crespo-Facorro, Kim, Andreasen, O'Leary, & Magnotta, 2000; Sanfilipo et al., 2000). Nevertheless, despite these methodological issues, the most frequent finding in the literature is an association between negative symptoms and morphometric alterations in the orbitofrontal cortex (OFC).

Most studies to date have involved patients with chronic Schizophrenia. Here, the severity of negative symptoms has been linked to alterations in GMV in the prefrontal cortex (Cascella et al., 2010; Galderisi et al., 2008; Koutsouleris et al., 2008), the temporal cortex (Galderisi et al., 2008; Koutsouleris et al., 2008; Sigmundsson et al., 2001), and limbic regions (Cascella et al., 2010; Sigmundsson et al., 2001), but these findings have not always been replicated (Arango et al., 2008; Moncrieff & Leo, 2010; Sanfilipo et al., 2000). A meta-analysis of studies of CTh in Schizophrenia reported that negative symptoms were associated with thinning in the left medial OFC, orbitofrontal gyrus and pars opercularis (Walton et al., 2018).

MRI studies in First Episode Psychosis (FEP) patients have linked negative symptoms with volumetric reductions and cortical thinning in the right parahippocampal gyrus (Benoit et al., 2012; Bodnar et al., 2014), thinning of the superior temporal, left orbitofrontal (Bodnar et al., 2014) and, right middle temporal cortex and increased CTh in the OFC (Makowski, Bodnar, Malla, Joober, & Lepage, 2016). However, as in the studies in chronic patients, these findings have not been consistently replicated (Crespo-Facorro et al., 2011; Fraguas, Diaz-Caneja, Pina-Camacho, Janssen, & Arango, 2016). To address the potentially confounding effects of previous antipsychotic treatment, some studies have examined FEP patients who were antipsychotic-naïve or minimally medicated. These studies have associated negative symptoms with reduced GMV in the left dorsolateral prefrontal (Ren et al., 2013) and inferior frontal cortex (Berge et al., 2011), and with increased thickness in the left total and lateral OFC (Lacerda et al., 2007), but thinning of the left medial OFC (Venkatasubramanian et al., 2008). Another study found no associations between negative symptoms and CTh (Xiao et al., 2015). A systematic review of the structural studies in high clinical risk for psychosis has linked negative symptoms with hippocampus, amygdala, corpus callosum, mPFC, and olfactory bulb.(Metzak, Devoe, Iwaschuk, Braun, & Addington, 2020). Overall, the most frequently reported findings have been in the left OFC, and to a lesser extent, the superior temporal cortex (Table 1).

**Table 1.** Summary of structural MRI studies investigating neural correlates of negative symptoms

Study	Patients/HC	Neuroimaging analysis method	Results
CHR for psychosis			
Metzak et al. (2020)	2144 (SR)	VBM/FS	Reduced GMV in Hippocampus, Amygdala, CC, MPFC, OB
Medication naïve			
Xiao et al. (2015)	128	FS	NS
Ren et al. (2013)	100	VBM	Reduced GMV R MTC Increased GMV DLPF
Lui et al. (2009)	68	VBM	Reduced GMV STG
Berge et al. (2010)	21	VBM	Reduced GMV IFC
Venkatasubramanian et al. (2008)	54	Cortical Thc Analysis	Reduced L MOFC
Lacerda et al. (2007)	43/54	VBM	Increased GMV L OFC, L Lateral OFC
Medicated patients – FEP			
Makowski et al. (2016)	97/48	Cortical Thc Analysis/ROI	Reduced R MTC Increased B OFC
Bodnar et al. (2014)	62/60	Cortical Thc Analysis	Reduced R STG, R PHC L OFC
Morch-Johnsen et al. (2015)	70	VBM	Reduced GMV L OFC, L ACC
Benoit et al. (2012)	16	VBM	Reduced GMV R PHC
Medicated patients – chronic			
Koutsouleris et al. (2008)	175/177	VBM	Reduced B OFC, L ACC, IFG
Galderisi et al. (2008) – *SzBull*	34	ROI	Reduced GMV R TLC
Cascella et al. (2010)	19/90	VBM	Reduced GMV: STG, SFG, L SMA, L ACC, L insula, L cuneus, R putamen.
Sigmundsson et al. (2001)	27/27	VBM	Reduced GMV: L STG, L ACC
Walton et al. (2018)	1985 (MA)	FS	Reduced CTh: L MOFC, L PO, L Lateral OFC
Different stages of schizophrenia spectrum			
Kirschner et al. (2021)	226/111	FS	Increased CTh L Lateral OFC (SPT) Increased CTh L Lateral OFC (MFEP) Reduced R Accumbens and B Putamen (SPT) Decreased CTh OFC (FEP) Increased B Putamen (SZ)

GMV, Grey Matter Volume; SR, Systematic Review; VBM, Voxel Based Morphometry; FS, FreeSurfer; CC, Corpus Callosum; MPFC, Medial Prefrontal Cortex; OB, Olfactory Bulb; NS, Non Significant; MTC, Middle Temporal Cortex; DLPF, Dorsolateral Prefrontal Cortex; STG, Superior Temporal Gyrus; IFC, Inferior Frontal Cortex; CTh, Corical Thickness; ROI, Region of Interest; L, Left; R, Right; B, Bilateral; OFC, Orbitofrontal Cortex; ACC, Anterior Cingulate Cortex, CG, Cingulate Gyrus; OFC, Orbitofrontal Cortex; PHG, Parahippocampal Gyrus; IFG, Inferior Frontal Gyrus, TLC, Temporal Lobe Cortex; SMA, Suplementary Motor Area, SFG, Superior Frontal Gyrus; MA, Meta-Analysis; MOFC, Medial Orbitofrontal Cortex; PO, Pars Opercularis; SPT, Schizotypal Personality Traits; MFEP, Medicated First Episode Psychosis.

Our aim was to examine concurrently GMV and CTh abnormalities in prefrontal and temporal brain regions previously associated with negative symptoms, in a large number of FES patients with Prominent Negative Symptoms (PMN), who had little or no exposure to antipsychotic treatment, by using the VBM and FreeSurfer (FS) software. We predicted that patients with PMN symptoms relative to those without, would have GMV and CTh alterations primarily in the OFC, the brain region most frequently linked to negative symptoms in published research to date (Lacerda et al., 2007; Makowski et al., 2016; Venkatasubramanian et al., 2008; Walton et al., 2018).

## Methods

Participants were 18–40 years old, and met DSM-IV criteria for the First Episode of Schizophrenia, Schizophreniform or Schizoaffective disorder, as defined using the Mini-International Neuropsychiatric Interview (Sheehan et al., 1998). They were either naïve to antipsychotic medication or had received <2 weeks of antipsychotic medication in the previous year and/or <6 months lifetime exposure. Patients were recruited over a 5-year period, as part of OPTiMiSE, a large multi-centre study of treatment response in Schizophrenia (www.optimisetrial.eu; EudraCT-Number: 2010-020185-19; clinicaltrials.gov identifier: NCT01248195). Exclusion criteria were an interval between the onset of psychosis and study entry >2 years, a need for coercive clinical care, and pregnancy. All data reported in the present study were collected prior to starting a clinical trial, which has been described in detail elsewhere (Kahn et al., 2018).

Ethical approval was obtained for each study centre from the local research ethics committee. All study participants provided written informed consent before entering the study and met safety criteria for MRI.

### Clinical assessments

Psychopathology of negative symptoms and overall severity of illness were assessed using the Positive and Negative Syndrome Scale (PANSS) (Kay, Fiszbein, & Opler, 1987) and the Clinical Global Impression scale (CGI) respectively. In the absence of the precise and universally accepted operational definitions of negative symptoms researchers to date have included differing sets of negative symptoms, incorporating both negative and general subscale items in various permutations. We have opted for the Liemburg Factor (Liemburg et al., 2013), that as our own factor analytic work in OPTiMiSE sample symptoms confirmed, best represents negative symptoms when PANSS is used [Bayesian Information Criterion (BIC) = 191.893; Root-Mean-Square-Error-of-Approximation (RMSEA) = 0.058, Comparative Fit Index (CFI) = 0.98] (Demjaha et al., 2018). The Liemburg Factor consists of 9 PANSS items: Flat Affect (N1), Emotional Withdrawal (N2), Poor Rapport (N3), Passive Social Withdrawal (N4), Lack of Spontaneity (N6), Mannerisms or Posturing (G5), Motor Retardation (G7), Avolition (G13), and Active Social Avoidance. (G16). We, therefore, used these items to produce a total negative symptom score. Patients were stratified into two subgroups: patients with Prominent Negative (PMN) symptoms (*N* = 88) and patients without Prominent Negative (non-PMN) Symptoms (*N* = 92), with the former defined as a total score ⩾20, based on the value of the originally derived scores from Kay et al., (Kay et al. 1987; Kay, Opler, & Lindenmayer, 1988) and employed in recently published research (Ren et al., 2013; Xiao et al., 2015).

### MRI acquisition

MRI data were acquired from 3 T scanners at eight sites (King's College London, UK; University Medical Centre Utrecht, The Netherlands; Mental Health Centre, Glostrup, Denmark, Sheba Medical Centre, Israel; University of Campania Luigi Vanvitelli (former University of Naples SUN), Italy: Fundación Cien, Sermas Madrid, Spain; Psychiatricka centrum Praha, Czech Republic; Orygen Youth Health, Australia): using the ADNI-2 protocol for multi-centre studies, www.loni.ucla.edu/ADNI/Research/Cores. Anonymised MRI images were transferred by individual scanning sites to a central database, using an encrypted file transfer protocol, for quality control and analysis.

### Image pre-processing and analyses

#### Voxel-based morphometry (VBM)

Imaging data were pre-processed and analysed by AD using MATLAB R2008b (The MathWorks Inc., Natick, MA, USA) and Statistical Parametric Mapping software (SPM12; The Wellcome Department of Imaging Neuroscience, London, UK; www.fil.ion.ucl.ac.uk/spm). The T1-weighted images were visually inspected for possible artefacts or any gross anatomical abnormalities by a single-researcher (AD), that could affect pre-processing, and aligned along the Anterior–Posterior Commissure. The images were pre-processed using the DARTEL (diffeomorphic anatomical registration through exponentiated lie algebra) (Ashburner, 2007), implemented in SPM12 toolbox. Each structural image underwent segmentation and the resultant grey matter and white matter images were used to generate an unbiased study-specific template. All data were then normalised to MNI space via this template to adjust for residual normalisation inaccuracies and anatomical variation. Following visual inspection for homogeneity across the sample, the GM images were smoothed with a 10 mm isotropic Gaussian kernel. The modulated, smoothed and normalised images then entered statistical analysis. In addition, Total Intracranial Volume (TIV) was calculated for all participants by GO using an in-house script developed by one of co-authors MK, which calculates and sums the total volume of grey matter, white matter and CSF using the maps derived from unified segmentation of the high-resolution T1-weighted image.

#### Freesurfer

Single T1-weighted images were automatically processed using default settings of FS (version 6.0.0; Massachusetts General Hospital, Harvard Medical School; http://surfer.nmr.mgh.harvard.edu) by AL, for cortical and subcortical reconstruction.

Reconstructed cortical surfaces for the left and right hemisphere were parcellated using the Desikan–Killiany atlas (Desikan et al., 2006) and subjected to a systematic Quality Assurance protocol (ENIGMA Cortical QC April 2017; http://enigma.usc.edu/protocols/imaging-protocols). As a part of this protocol multiple internal and external views of the reconstruction were visually inspected and rated by a single-rater (AD). Two participants were excluded from this analysis because of inadequate data quality following the reconstruction process.

#### Region of interest (ROI) analysis

Region-of-interest (ROI) analyses of the VBM data were performed in bilateral prefrontal and temporal brain regions previously associated with negative symptoms (Cascella et al., 2010; Chua et al., 1997; Crespo-Facorro et al., 2011; Fraguas et al., 2016; Galderisi et al., 2008; Koutsouleris et al., 2008; Sanfilipo et al., 2000; Shenton, Dickey, Frumin, & McCarley, 2001; Walton et al., 2018): the superior orbitofrontal, medial orbitofrontal, inferior orbitofrontal and superior temporal gyri. A single anatomical mask that included these eight ROIs was created using the AAL Human Atlas (via the WFU PickAtlas toolbox). We then applied a Small Volume Correction (SVC) using this mask, setting significance level at *p* < 0.05, after Family-Wise Error (FWE) correction for multiple comparisons, in SPM-12. To allow for the homogeneity of the sample across different analyses, we have repeated VBM analysis after removing two participants excluded from the FS analysis

#### Exploratory whole-brain analyses

In addition to ROI analysis, VBM and FS were employed to explore whether there were group differences at the whole-brain level. VBM was used to run voxel-wise comparisons using an ANCOVA design, with significance set at *p* < 0.05, after FWE correction for multiple comparisons. CTh was examined using FS version 6.00, QDEC (Query, Design, Estimate Contrast) tool, providing a measure of thickness across the cortical surface of the whole brain, using the General Linear Model (GLM). Surface-based group analysis was computed to conduct vertex-by-vertex comparisons for each hemisphere. Prior to analysis CTh maps were transformed into a common space (the freesurfer *fsaverage* surface) and smoothed with a full width at half maximum Gaussian kernel of 10 mm. Between-group differences were corrected for multiple comparisons via Monte Carlo z-field simulation at *p* *<* *0.05* (two-tailed).

For all MRI analyses, site, age and TIV were used as covariates. To control for site effects, we used dummy-coded site variables. Because patients with PMN, in addition to higher negative symptom severity, also had more severe positive and general symptoms, and higher CGI scores, compared to the non-PMN patients, we examined that the group effect was not influenced by these other clinical features. To do this we extracted volume estimates using MarsBar (http://marsbar.sourceforge.net/about.htm) in clusters where significant between-group differences in GMV were identified, and similarly, we extracted the CTh in regions where there were significant differences between groups. We then conducted partial correlations between the respective measures from these regions and positive and general symptoms, as well as Illness severity, whilst co-varying for age, TIV and site.

## Results

The PMN and non-PMN groups did not differ significantly in terms of sex, ethnicity, or scanning site (Table 1). However, the PMN group were relatively younger, had a lower educational level, and a greater proportion of patients had a diagnosis of Schizophrenia, as opposed to Schizophreniform or Schizoaffective psychosis. The PMN group also had higher scores on the CGI and all of the PANSS subscales (Table 2).

**Table 2.** Sociodemographic and clinical characteristics of the sample

	PMN (*N* = 88)	non-PMN (*N* = 92)	Statistics
Age years, mean [s.d.]	23.61 [5.3]	25.75 [5.6]	*t* = 2.6; *p* = 0.01
Sex, *N* [%]			χ^2^ = 0.35; *p* = 0.63
Male	62 [70.5]	61 [66.3]	
Female	26 [29.5]	31 [33.7]	
Diagnosis, *N* [%]			χ^2^ = 14.32; *p* = 0.01
Schizophrenia	49 [55.7]	38 [45.8]	
Schizoaffective	0 [0]	5 [13.2]	
Schizophreniform	39 [44.3]	49 [41.0]	
Antipsychotic-naïve, *N* [%]			χ^2^ = 0.06; *p* = 0.81
Yes	51 [58.0]	55 [59.8]	
No	37 [42.0]	37 [40.2]	
Previous antipsychotic, *N* AM/AR/OL/RI/HA	26/2/5/2/2	20/2/5/9/1	χ^2^ = 6.3; *p* = 0.39
Previous AP duration, days: median, (min–max)	8 (2–14)	9 (2–14)	
Previous antipsychotic dose (mg), mean [s.d.]			
Amisulpiride	471.15 [275.02]	437.5 [314.93]	
Aripiprazole	30 [0.0]	10 [7.1]	
Olanzapine	9 [2.2]	9 [2.2]	
Risperidone	2 [1.4]	3 [1.5]	
Concomitant medication MS/BDZ/AD/S/H	2/7/9/1/0	4/18/3/1/1	χ^2^ = 6.3; *p* = 0.08
Ethnicity, *N* [%]			χ^2^ = 2.35; *p* = 0.49
White	76 [86.4]	73 [79.3]	
Other	12 [13.0]	19 [20.7]	
Education years, mean [s.d.]	11.43 [2.37]	12.51 [3.27]	*t* = 2.6; *p* = 0.01
Study centre (*n*) UK/CZ/DK/IL/IT/ES/NL/AU	9/1/21/24/12/14/6/1	14/3/9/24/14/19/7/2	χ^2^ = 0.93; *p* = 0.3
CGI severity	5.5 [0.9]	5.0 [0.9]	*t* = 3.3; *p* = 0.001
PANSS total score mean [s.d.]	83.0 [13.6]	60.3 [11.1]	*t* = 12.3; *p* = 0.001
PANNS negative subscale mean [s.d.]	22.3 [4.5]	12.1 [3.5]	*t* = 17.2; *p* = 0.001
PANSS positive subscale mean [s.d.]	20.5 [5.6]	17.37 (4.6)	*t* = 4.1; *p* = 0.001
PANNS general subscale mean [s.d.]	40.3 [9.8]	30.9 [6.8]	*t* = 8.5; *p* = 0.001

Study Centre: UK, United Kingdom; CZ, Czech Republic; DK, Denmark; IL, Israel; IT, Italy; ES, Spain; NL, Netherlands; AU, Australia. Previous Antipsychotic: AM, Amisulpiride; AR, Aripiprazole; OL, Olanzapine; RI, Risperidone; HA, Haloperidol. Concomitant Medication: MS, Mood Stabilizer; BDZ, Benzodiazepines; AD, Antidepressant; S, Stimulant; H, Hypnotic.

### Voxel-based morphometry (VBM)

**Fig. 1.**
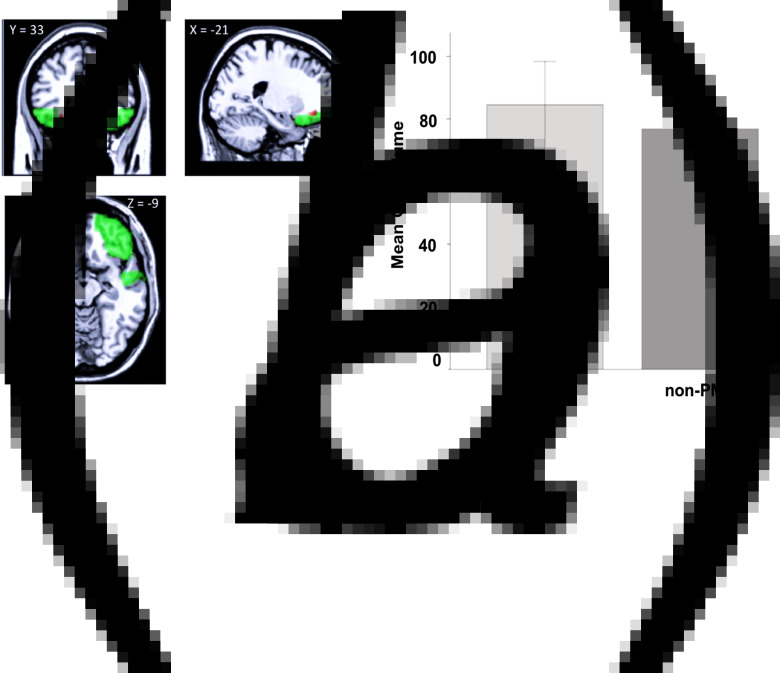
(*a*) Brain coronal, sagittal and axial sections demonstrating contrasts between PMN and non-PMN patients. Red: larger left inferior OFC in PMN relative to non-PMN patients. Green: anatomical ROI mask [MNI (Montreal Neurological Institute) coordinates *x*, *y*, and *z*: −21, 33, and 9, respectively, PFWE-corrected = 0.03; *z* = 4.01; and cluster size = 34 voxels]. For illustrative purposes only, results are displayed at *p* < 0.005 after FWE correction for multiple comparisons. (*b*) The plot shows mean GM volumes for the significant cluster within the left Inferior OFC in cubic millimetres per voxel, for the 2 groups. Error bars represent s.d.

The ROI analysis revealed that the left inferior orbitofrontal volume was larger in the PMN than the non-PMN group. This difference remained significant after applying SVC and controlling for age, TIV and site [MNI (Montreal Neurological Institute) coordinates *x*, *y*, and *z*: −21, 33, and −9, respectively, _FWE-corrected_ = 0.03; *z* = 4.01; and cluster size = 34 voxels] (Fig. 1). There were no significant differences in the other ROIs. After removing two participants excluded from the FS analysis, the results remained significant: MNI coordinates *x*, *y*, and *z*: −20, 33, and −9, respectively, P_FWE-corrected_ = 0.03; *z* = 4.02; and cluster size = 31 voxels. In the whole-brain VBM analysis, there were no significant group differences, even at an uncorrected threshold.

#### Freesurfer (QDEC) whole-brain analysis

**Fig. 2.**
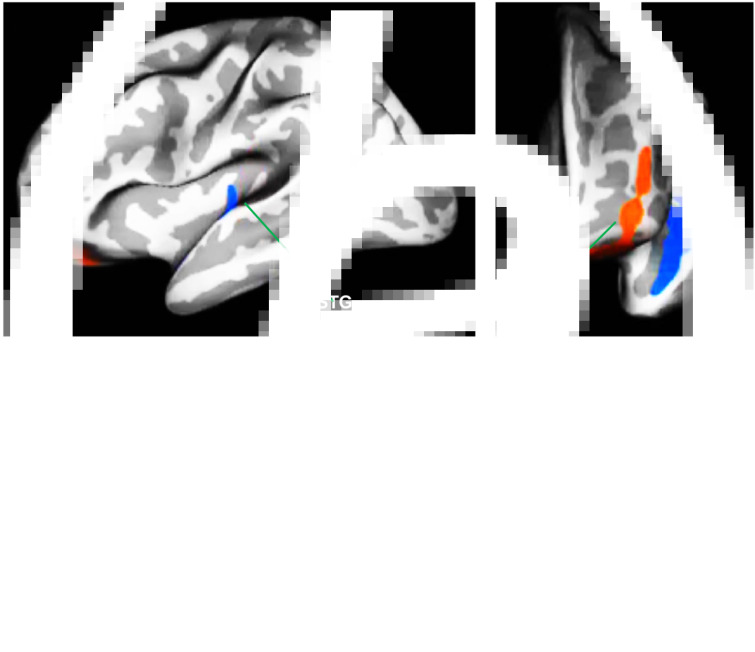
Significant cortical alterations projected onto the inflated surface of the left hemisphere in (*a*) lateral and (*b*) frontal views. Cortical thickness (CTh) was significantly increased in the OFC and decreased in the superior temporal gyrus (STG) in PMN compared to the non-PMN group following correction for multiple comparisons using Monte Carlo simulation, *p* < 0.05. OFC is shown in red and STG in blue.

Two participants were excluded from this analysis because of inadequate data quality following the reconstruction process. CTh was greater in the left lateral OFC in the PMN than in the non-PMN group. Conversely, in the left superior temporal gyrus, the cortex was thinner in the PMN than in the non-PMN group (Fig. 2).

### Correlations between MRI measures and positive and general symptoms, and illness severity

Left Inferior OFC GMV was not correlated with the severity of either positive [*r*(169) = 0.09, *N* = 180, *p* = 0.3] or general symptoms [*r*(169) = 0.13, *N* = 180, *p* = 0.1], or with CGI scores [*r*(169) = −0.006, *N* = 180, *p* = 0.9]. Similarly, there were no correlations between CTh in the left OFC and left STG and the severity of positive [*r*(167) = −0.06, *N* = 178, *p* = 0.4; *r*(167) = −0.1, *N* = 180, *p* = 0.2 respectively], or general symptoms [*r*(167) = −0.03, *N* = 178, *p* = 0.7; *r*(167) = 0.06, *N* = 178, *p* = 0.9 respectively], or CGI scores [*r*(167) = −0.11, *N* = 178, *p* = 0.1; *r*(167) = −0.13, *N* = 178, *p* = 0.08 respectively].

### Correlations between MRI measures and negative symptoms

Across all subjects (in both groups), left inferior OFC volume [*r*(169) = 0.16, *p* = 0.03], left lateral OFC thickness [*r*(167) = −0.2, *p* = 0.01] and left superior temporal CTh [*r*(167) = −0.18, *p* = 0.02] were all significantly correlated with PANSS negative symptom scores.

## Discussion

To our knowledge, this is the largest neuroimaging study to date to concurrently investigate GMV and CTh in temporal and prefrontal regions in patients with PMN symptoms. We examined patients that were homogeneous for a stage of illness, and were antipsychotic-naïve or minimally treated.

Our main findings were that patients with PMN symptoms had greater left inferior OFC volume, greater thickness of the left lateral OFC, and a thinner left superior temporal cortex. These results are consistent with those from previous studies that have linked negative symptoms in Schizophrenia with alterations in the orbitofrontal and superior temporal cortex (Baare et al., 1999; Bodnar et al., 2014; Koutsouleris et al., 2008; Lacerda et al., 2007; Venkatasubramanian et al., 2008). These regions play a crucial role in social cognition and are considered to be an integral part of the ‘social brain’ (Allison, Puce, & McCarthy, 2000; Zilbovicius et al., 2006). Therefore, the findings suggest that negative symptoms may reflect a disruption of these functions (Chemerinski et al., 2002; Gur et al., 2000). The OFC has intricate interconnections with the superior temporal lobe via which the role in the pathophysiology of negative symptoms may be mediated (Zald & Kim, 2008). The finding that OFC may be implicated in the pathophysiology of negative symptoms is not surprising. Orbitofrontal lesions can lead to apathy, lack of drive, social withdrawal and blunted affect in humans (Blumer, 1975; Grafman, Vance, Weingartner, Salazar, & Amin, 1986), and to social withdrawal in non-human primates (Raleigh & Steklis, 1981), which all reflect negative symptomatology.

In the present study, we found that negative symptoms were linked to *increased* orbitofrontal CTh and volume. Several previous MRI studies in patients with chronic Schizophrenia have associated negative symptoms with *reductions* in thickness and volume in this region (Bodnar et al., 2014; Koutsouleris et al., 2008; Morch-Johnsen et al., 2015; Walton et al., 2018).^.^ Conversely, studies in FEP patients have reported that negative symptoms were associated with *greater* CTh and volume in the left inferior and lateral OFC (Lacerda et al., 2007; Makowski et al., 2016) as in the present study. In general, MRI studies in FEP patients often describe volumetric increases in regions that show volumetric reductions in chronic Schizophrenia (Lacerda et al., 2007; Ren et al., 2013; Xiao et al., 2015). Differences in the findings in chronic and first-episode samples could reflect effects of illness chronicity and/or antipsychotic medication on MRI measures (Ho, Andreasen, Ziebell, Pierson, & Magnotta, 2011; Moncrieff & Leo, 2010). In addition, the nature of morphometric alterations in Schizophrenia may depend on the neurodevelopmental stage of the patient at the time of scanning, particularly if brain maturation, neural migration and synaptic pruning are altered in those with the disorder (Keshavan, Anderson, & Pettegrew, 1994; Keshavan & Hogarty, 1999; Lacerda et al., 2007). Another possibility is that increases in CTh or volume may occur at the first episode stage as part of an initial compensatory response to the development of psychosis that is no longer active in the chronic phase (Goghari, Rehm, Carter, & MacDonald, 2007).

We also found that negative symptoms were associated with cortical thinning in the left superior temporal gyrus. Bodnar and colleagues (Bodnar et al., 2014) observed a similar association with cortical thinning in this region, however in the right hemisphere. Although negative symptoms have been linked to alterations in superior temporal GM volume, (Cascella et al., 2010; Lui et al., 2009; Sigmundsson et al., 2001), it is of note that the STG alterations are not specific to negative symptomatology. Volumetric reductions of the left STG have been linked with positive symptoms, (Koutsouleris et al., 2008) and in particular auditory hallucinations (Nenadic, Sauer, & Gaser, 2010). In the present study, we did not find evidence of GMV alterations in STG. These discrepant and unexpected findings within the same sample could be due to the computational differences between the two image analyses techniques. Surface-based analysis employing FS software measures the CTh in millimetres, whereas VBM measures GM differences in local surface area and cortical folding. Furthermore, in accordance with previous scientific reports the fact that by using FS we detected another regional association suggests that measures of CTh may be more sensitive than VBM and thus complementary when defining GM anomalies (Bodnar et al., 2014). The cortical thinning in the left superior temporal gyrus nonetheless complements the finding of GM abnormalities in OFC. Like the OFC, the superior temporal cortex is implicated in social perception and cognition (Allison et al., 2000; Zilbovicius et al., 2006), and the two regions are densely interconnected, particularly within the same hemisphere (Zald & Kim, 2008).

It is also of interest that our findings are lateralised to the left brain hemisphere. Although studies investigating neural correlates of negative symptoms have identified bilateral alterations, most findings have been pertained to the left hemisphere (see Table 1). Since the left hemisphere typically specialises in language production and emotional processing (Griggs, 2012), we speculate that left hemisphere functioning may be affected in patients with negative symptoms of Schizophrenia, however this is yet to be established in the future scientific work.

### Strengths and limitations

Our study has several strengths: (a) it is the largest studies of the neural correlates of negative symptoms in antipsychotic-naïve or minimally treated patients, (b) both CTh and GMV were examined using state of the art neuroimaging methods that provided more information about cortical architecture involved in negative symptoms (Palaniyappan & Liddle, 2012), (c) All participants had a Schizophreniae-spectrum psychosis, (d) participants were scanned using the same MRI methodology, (c) the set of PANSS negative symptoms that we examined was validated in confirmatory factor analytic work (Liemburg et al., 2013; Stiekema et al., 2016) including our own conducted in the same patient sample (Demjaha et al., 2018).

In order to maximise the sample size, we acquired MRI data from multiple centres. Although we employed an MRI protocol (ADNI) designed for multi-site studies, and controlled for a site in the analysis, we might have further reduced site effects by scanning a group of the same volunteers at each of the different centres, and comparing the data from each site. Although two groups differed on the severity of illness and positive symptom scores, the absence of significant correlations between identified significant brain regions with these variables, determined that the findings are unrelated to these variables. Finally, due to QDEC methodological restrictions, we were unable to restrict our FS analysis to ROIs as we did in VBM, however the whole-brain analysis of CTh revealed significant changes in the hypothesised regions. Another potential limitation may relate to the fact that some of our patients have been minimally treated with antipsychotics, however the groups did not differ in the mean dose of medication used. In addition, we may have included patients who were less severely ill compared to the general FEP population, as we excluded patients receiving compulsory treatment and included those willing to participate in a clinical trial. Finally, we could have assessed negative symptoms using an instrument that is specific for the evaluation of these symptoms. However, while these may provide a better assessment than general scales like the PANSS, they take longer to administer and may require extensive training, making them less practicable in a large study involving multiple different sites. In addition, the PANSS is one of the most frequently used rating scale in clinical trials (Malaspina et al., 2014), potentially increasing the generalisability of findings.

## Conclusions

Our results highlight the role of the alterations in the orbitofrontal and superior temporal cortex in the pathophysiology of negative symptoms in Schizophrenia. There is a pressing need for new treatments for negative symptoms: understanding their neurobiological basis may inform the development of novel therapeutic approaches. The OFC in particular may be a significant biomarker for designing effective clinical trials for negative symptoms. Future studies employing longitudinal designs as well as functional imaging investigating patients from ultra-high risk states to established pre-treatment psychotic illness, are needed to further elucidate and confirm OFC as a potential target for novel treatment developments.
